# Ultrafast electron transfer kinetics at semiconductor-microbe interface: key to efficient extracellular photoelectron utilization

**DOI:** 10.1128/aem.00138-25

**Published:** 2025-07-31

**Authors:** Yimei Du, Yan Li, Yanzhang Li, Song Jin, Huan Ye, Bingxu Hou, Tianci Hua, Jiaqi Zhu, Houze Lu, Anhuai Lu, Tao Li

**Affiliations:** 1 SKLab-DeepMinE, MOEKLab-OBCE, School of Earth and Space Sciences, Peking University 420740 , Beijing, China; 2 Beijing Key Laboratory of Mineral Environmental Function, School of Earth and Space Sciences, Peking University 420740 , Beijing, China; 3 Department of Civil and Architectural Engineering, University of Wyoming 195931, Laramie, Wyoming, USA; 4 Key Laboratory of Algal Biology, Institute of Hydrobiology, Chinese Academy of Sciences 53021, Wuhan, China; Washington University in St. Louis, St. Louis, Missouri, USA

**Keywords:** semiconductor-microbe interface, extracellular photoelectron utilization, ultrafast electron transfer, azo decolorization, interfacial kinetics

## Abstract

The key limiting factors on energy conversion efficiency in semiconductor-microbe hybrid systems remain inadequately understood. This study investigates the impact of ultrafast electron transfer kinetics at Cd*_x_*Zn_1-_*_x_*S/*Shewanella oneidensis* MR-1 interfaces on the overall efficiency of hybrid systems. The reduction efficiency of direct blue 71 dye by Cd*_x_*Zn_1-_*_x_*S/MR-1 is significantly enhanced under light, leveraging the synergy of extracellular photoelectron transfer in various Cd*_x_*Zn_1-_*_x_*S nanoparticles and associated redox pathways in MR-1. Notably, CdS/MR-1 achieved a 98% reduction compared to 31% for ZnS/MR-1 after 1 hour, despite ZnS exhibiting a more favorable conduction band potential of −1.37 V vs normal hydrogen electrode (NHE). Time-resolved spectra and density functional theory calculations reveal that the efficiency advantages of CdS/MR-1 are attributed to its longer photoelectron lifetime (1.14 ± 0.12 ps vs 0.18 ± 0.03 ps for ZnS/MR-1) and higher electron mobility (119.71 cm²/V·s for CdS/MR-1 vs 62.47 cm²/V·s for ZnS/MR-1), providing MR-1 with superior kinetic advantages in utilizing photoelectron energy. Additionally, experiments with exogenous cytochrome *c* demonstrate its crucial role in modulating extracellular and intracellular electron transfer kinetics at the Cd*_x_*Zn_1-_*_x_*S/MR-1 interface. Transcriptomic analysis reveals similar photoelectron transfer pathways in CdS/MR-1 and ZnS/MR-1, supporting that CdS/MR-1′s superior efficiency stems from kinetic advantages at the interface, leading to greater bioavailable photoelectron accumulation. These findings underscore the importance of optimizing photoelectron transfer kinetics to enhance extracellular photoelectron utilization efficiency in semiconductor-microbe hybrid systems.

**IMPORTANCE:**

The synergy of light-sensitive semiconductor elements (e.g., natural minerals) and microbes in natural matrices enhances biological functions and opens a wide range of biotechnological possibilities. Given the extremely short lifetimes of photoelectrons and rapid transfer rates at semiconductor-microbe interface, understanding this ultrafast electron transfer process is essential for elucidating the mechanism of extracellular photoelectron utilization and optimizing system efficiency. Our study demonstrates that *Shewanella oneidensis* MR-1 can benefit from photoelectrons through ultrafast electron transfer pathways, similar to photosynthetic systems. For microbes to efficiently utilize these photoelectrons before charge recombination on an ultrafast timescale, a prolonged photoelectron lifetime is kinetically advantageous. Our findings indicate that the superior efficiency of the CdS/MR-1 hybrid system is driven by kinetic advantages rather than thermodynamic factors. This foundational study is crucial for optimizing the energetics of semiconductor-microbe hybrid systems and expands our understanding of microbial energy metabolism.

## INTRODUCTION

Solar energy, a vital sustainable energy source, drives the photoelectric effect in photosynthesis (1), semiconducting minerals (2), and organic matters (3). The high quantum efficiency of natural photosynthesis has inspired the development of semiconductor-microbe hybrid systems (4–6), which integrate light-sensitive semiconductor elements with microbes to harness solar energy for efficient pollutant degradation and sustainable energy storage in environmental contexts (7–9). Given the widespread of semiconducting minerals and electroactive microbes on Earth, microbial extracellular solar-driven photoelectron transfer is believed to be prevalent in sunlit soils, euphotic aquifers, and sulfide-rich early marine environments (10, 11), underscoring its significant potential in environmental applications. However, the energetic strategies and mechanisms by which microbes utilize photoelectron remain largely unexplored, requiring further research for the optimization of semiconductor-microbe hybrid systems.

Given that the non-photosynthetic bacterial envelope is neither physically permeable to most semiconductors nor electrically conductive, the extracellular electron transfer (EET) capability is essential for non-photosynthetic bacteria to serve as electron relays at the semiconductor-microbe interface. For example, *Shewanella oneidensis* MR-1 (MR-1) is a model organism for EET research. The metal reduction pathway (Mtr) is critical for bidirectional electron transfer in MR-1, facilitated by a set of electroactive membrane proteins, including OmcA, MtrC, MtrA, MtrB, MtrF, CymA, etc. (12). The thermodynamic mechanism concerning energy level alignment between the band edges of semiconductors and various microbial electron carries (e.g., redox shuttles and electroactive membrane proteins) has been a primary focus in semiconductor-microbe hybrid systems (13, 14). It is suggested that photoelectrons at higher energy levels in semiconductors present a thermodynamic superiority for microbial utilization (15). If electron transfer at the semiconductor-microbe interface were governed solely by thermodynamic principles, semiconductors with more negative potentials such as GaP (−1.5 V vs NHE) would be ideal for coupling with microbes (13). However, in practical applications, semiconductors with moderate redox potentials like CdS (−0.74 vs NHE) are predominantly employed in various semiconductor-microbe hybrid systems for processes such as hydrogen production, carbon dioxide fixation, and pollutant degradation (16). This inconsistency indicates the presence of unidentified mechanisms that influence the system efficiency beyond thermodynamic considerations.

In light energy-related systems, the kinetics of charge-carrier (photoelectron or photohole) process at interfaces, such as separation, recombination, and transfer, are pivotal in determining energy conversion efficiency. For instance, in photosynthetic system, charge recombination is minimized by efficient charge separation across a sophisticated arrangement of photosynthetic components like light harvesting antenna (e.g., P680 and P700) and electron relay (e.g., chlorophyll and quinones), ensuring lifetimes sufficient to drive water oxidation (17, 18). Similarly, the kinetic competition between charge-carrier recombination and transfer to microbes at the semiconductor-microbe interface is a major efficiency-limiting factor (19), which includes a series of steps occurring on ultrafast timescales, ranging from femtoseconds (fs, 10^−15^ s) to microseconds (μs, 10^−6^ s) (20). The transition of electrons in photoexcited semiconductors from the filled valence band (VB) to the empty conduction band (CB) occurs within fs, and the lifetime of electrons in the excited state spans only a few nanoseconds (ns, 10^−9^ s) (21). Therefore, extracellular photoelectron transfer must proceed on a comparable ultrafast timescale.

At the semiconductor-microbe interface, the kinetics of ultrafast photoexcited electron transfer processes can now be explored using time-resolved spectroscopic techniques (22). For instance, Kornienko et al. (23) employed transient absorption (TA) spectroscopy to study charge transfer kinetics from CdS to *Moorella thermoacetica*, revealing photoelectron transfer to hydrogenase within a few picoseconds (ps, 10^−12^ s) for the reduction of CO_2_ to acetic acid with high efficiency. Similarly, Hu et al. (24) showed that in an Au-cyanobacteria hybrid system, the photoelectron transfer to the potential electron acceptor in photosystem II occurs with a few ps to drive the reduction of CO_2_ to glycerol with improved efficiency. Furthermore, Shen et al. (25) indicated that reduced graphene oxide can extend the charge transfer timescale between *S. oneidensis* MR-1 and Cu_2_O nanosheets to several ns, enhancing hydrogen production. From a kinetic standpoint, to align with the intracellular electron transfer timescale (μs ~ ms) (26), the relaxation time of photoelectron in their excited states should be prolonged as much as possible, thereby enhancing the efficient utilization of photoelectron by microbes. This crucial aspect is often overlooked in optimizing the overall efficiency of hybrid systems and deserves in-depth investigation.

Therefore, this work focuses on the ultrafast electron transfer kinetics at the semiconductor-microbe interface. Considering the thermodynamic favorability of CdS and ZnS and the chemical similarity between Cd^2+^ and Zn^2+^, we utilized MR-1 for the *in situ* biosynthesis of Cd*_x_*Zn_1-_*_x_*S to develop the Cd*_x_*Zn_1-_*_x_*S/MR-1 hybrid system. Based on previous studies (27–29), azo dye decolorization can be used as an indicator to assess the apparent electron transfer efficiency of semiconductor-microbe hybrid systems. Accordingly, azo dye direct blue 71 (DB71) was employed in this study to evaluate the overall efficiency of Cd*_x_*Zn_1-_*_x_*S/MR-1 hybrid system, aiming to further explore the mechanism of efficient extracellular photoelectron utilization. Building on previous studies, we further concentrated on the ultrafast photoelectron transfer at the Cd*_x_*Zn_1-_*_x_*S/MR-1 interface and identified the key kinetics factors influencing the DB71 reduction efficiency using photoluminescence (PL) spectra, time-resolved PL (TRPL) spectra, and TA spectra (TAS), in conjunction with density functional theory calculations and transcriptomic analysis.

## RESULTS AND DISCUSSION

### Construction of Cd*_x_*Zn_1-_*_x_*S/MR-1 hybrid systems

**Fig 1:**
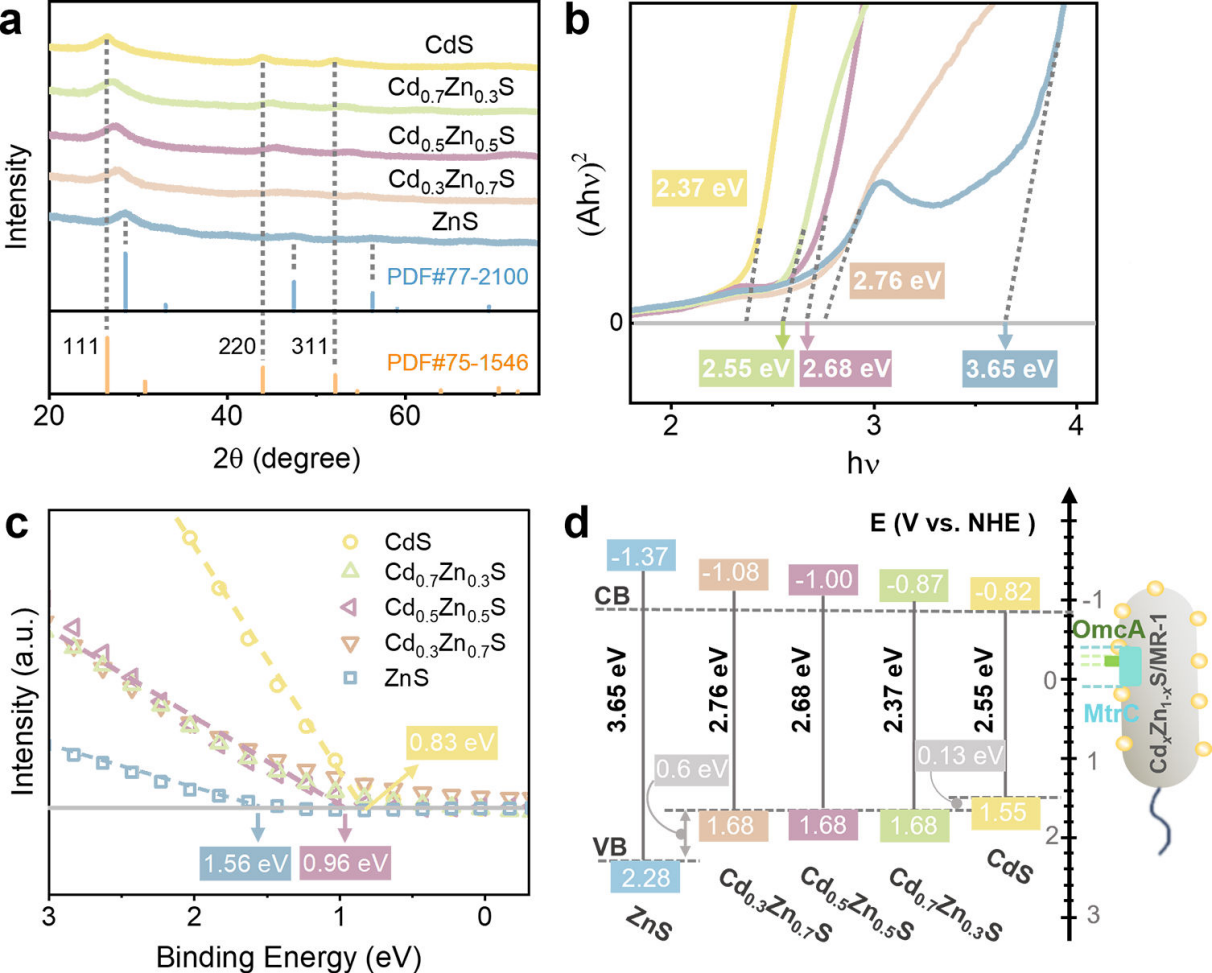
Characterization of Cd*_x_*Zn_1-_*_x_*S/MR-1 hybrid system. (**a**) XRD patterns recorded in the range of 20–75 (2θ°). (**b**) Bandgap estimation using Tauc plot. (**c**) Valence-band XPS analysis relative to Fermi energy level (0 eV). (**d**) A diagram of redox potentials for CB and VB positions of Cd*_x_*Zn_1-_*_x_*S, and thermodynamic redox potentials for key electroactive proteins of MR-1. Each experiment was repeated three times.

Schematic and actual images of Cd*_x_*Zn_1-_*_x_*S/MR-1 hybrid system as shown in Fig. S1. After 48 h construction of Cd*_x_*Zn_1-_*_x_*S/MR-1 hybrid system, we measured the concentration of Cd^2+^ and Zn^2+^ in solution and found that it was below the detection limit. This indicates that no free Cd^2+^ and Zn^2+^ remained, and all were incorporated into the Cd*_x_*Zn_1-_*_x_*S during biosynthesis. The X-ray diffraction (XRD) patterns (Fig. 1a) and transmission electron microscopy (TEM) images (Fig. S2) confirmed the successful synthesis of cubic Cd*_x_*Zn_1-_*_x_*S nanoparticles, which were distributed around the MR-1 cells with close contact. By measuring the Cd^2+^ and Zn^2+^ concentrations of Cd*_x_*Zn_1-_*_x_*S solids (Table S1), the Cd*_x_*Zn_1-_*_x_*S samples were identified as CdS, Cd_0.7_Zn_0.3_S, Cd_0.5_Zn_0.5_S, Cd_0.3_Zn_0.7_S, and ZnS. As the decreased radius of cations from 0.78 Å for Cd^2+^ to 0.60 Å for Zn^2+^ caused the shrinkage of unit cells, leading to the shift of diffraction peaks with the same miller index to higher angle in XRD patterns with increasing proportion of Zn^2+^ (Fig. 1a). The ultraviolet-visible (UV-vis) absorption spectra (Fig. S3) and their Tauc plots (Fig. 1b) indicated that the bandgaps of the Cd*_x_*Zn_1-_*_x_*S samples from CdS to ZnS were 2.37, 2.55, 2.68, 2.76, and 3.65 eV, respectively. Furthermore, the relative energy positions of VB edge of Cd*_x_*Zn_1-_*_x_*S samples were obtained by measuring the valence-band X-ray photoelectron spectroscopy (XPS) analysis (Fig. 1c). Through straight-line extrapolation, the VB edge of Cd*_x_*Zn_1-_*_x_*S relative to Fermi level (E_f_) was determined to be 0.83, 0.96, 0.96, 0.96, and 1.56 eV for CdS, Cd_0.7_Zn_0.3_S, Cd_0.5_Zn_0.5_S, Cd_0.3_Zn_0.7_S, and ZnS, respectively. This suggested that the VB positions of Cd_0_._7_Zn_0_._3_S, Cd_0.5_Zn_0.5_S, and Cd_0_._3_Zn_0_._7_S are similar, while those of CdS and ZnS are 0.13 eV higher and 0.60 eV lower than the middle three samples, respectively. The CB energy difference between ZnS and CdS is 0.55 eV, close to the reported value of 0.52 eV (30). The absolute CB energy levels are −3.46 eV for ZnS and −3.98 eV for CdS (30). Due to the increasing bandgap with the incorporation of Zn^2+^, a higher Zn^2+^ content in Cd*_x_*Zn_1-_*_x_*S samples resulted in a more negative CB energy (Table S2; Fig. 1d). At pH = 7, the potentials of CB and VB for ZnS are 2.28 and −1.37 V (vs NHE), respectively, while those for CdS are 1.55 and −0.82 V (vs NHE), respectively.

The cell viability and percentage of live bacteria of Cd*_x_*Zn_1-_*_x_*S/MR-1 hybrid systems were assessed by using the CCK-8 assay and N,N-dimethylaniline N-oxide (DMAO)/propidium iodide (PI) staining test (Fig. S4), demonstrating that MR-1 maintained normal viability. This confirmed the effective construction of a series of Cd*_x_*Zn_1-_*_x_*S/MR-1 hybrid systems with light-sensitive semiconductor elements and good cell viability, facilitating further investigation into ultrafast electron transfer at the semiconductor-microbe interface. From a thermodynamic perspective, as shown in Fig. 1d, the transfer of photoelectron from the CB of Cd*_x_*Zn_1-_*_x_*S (potentials ranging from −0.82 to −1.37 V vs NHE at pH 7) to the critical extracellular electron uptake membrane proteins of MR-1, such as MtrC (−0.40 ~ 0.10 V vs NHE, pH 7), OmcA (−0.32 ~ −0.20V vs NHE, pH 7), and NAD^+^/NADH (−0.32V vs NHE, pH 7), is thermodynamically feasible (31, 32). The more negative CB position of ZnS compared to CdS suggests a superior reduction capacity and a greater thermodynamic advantage for ZnS in interfacial electron transfer.

### Overall efficiency of Cd*_x_*Zn_1-_*_x_*S/MR-1 hybrid systems

**Fig 2:**
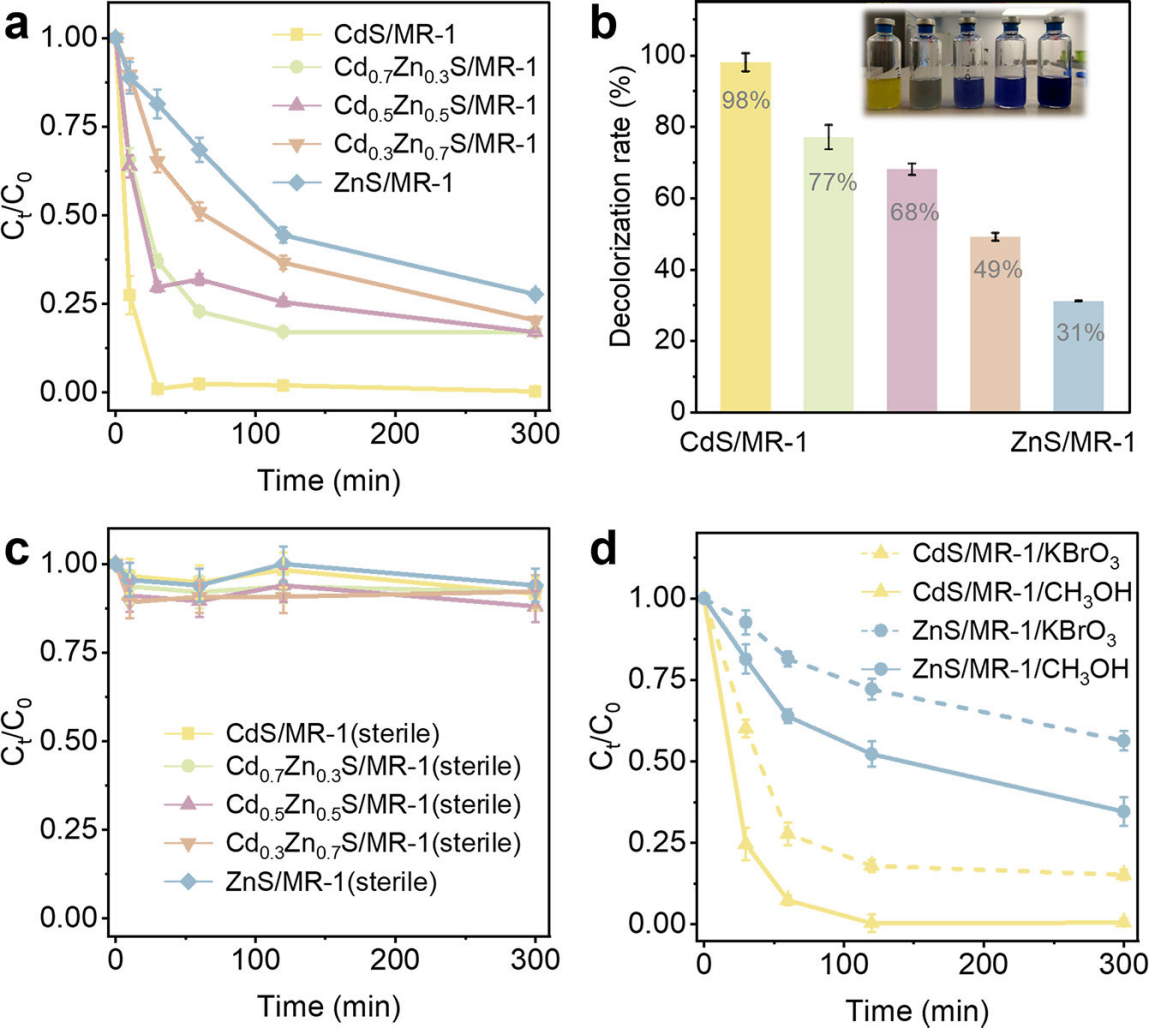
Azo dye decolorization performance of Cd*_x_*Zn_1-_*_x_*S/MR-1 hybrid system. (**a**) Decolorization curves of DB71 over time for Cd*_x_*Zn_1-_*_x_*S/MR-1 systems. (**b**) Average decolorization efficiency of various Cd*_x_*Zn_1-_*_x_*S/MR-1 systems after one hour. (**c**) Decolorization curves of DB71 over time for sterile Cd*_x_*Zn_1-_*_x_*S/MR-1 systems. (**d**) Decolorization curves of DB71 over time with the addition of KBrO_3_ or CH_3_OH. Each experiment was repeated three times.

Under identical irradiation conditions (Fig. 2a and b), the decolorization efficiency of DB71 for CdS/MR-1, Cd_0.7_Zn_0.3_S/MR-1, Cd_0.5_Zn_0.5_S/MR-1, Cd_0.3_Zn_0.7_S/MR-1, and ZnS/MR-1 was determined to be 98%, 77%, 68%, 49%, and 31% after 1 hour, respectively. The efficiency decreased with increasing Zn concentration, with CdS/MR-1 being three times more efficient than ZnS/MR-1. In contrast, sterile Cd*_x_*Zn_1-_*_x_*S/ MR-1 all removed less than 10% of DB71 (Fig. 2c), underscoring the limited photocatalytic efficiency of Cd*_x_*Zn_1-_*_x_*S alone and the critical role of MR-1 cell viability for efficient electron transfer. According to the control experiments (Fig. S5), it was found that DB71 could be slowly decolorized by MR-1, achieving about 30% decolorization efficiency within 5 hours. In the absence of either Cd*_x_*Zn_1-_*_x_*S or MR-1, DB71 could not be photoreduced. Obviously, all Cd*_x_*Zn_1-_*_x_*S/MR-1 systems achieved enhanced decolorization efficiency compared to MR-1 under identical irradiation conditions. It is demonstrated the synergy of MR-1 and photoexcited semiconducting Cd*_x_*Zn_1-_*_x_*S for enhanced decolorization.

To elucidate the contributions of photoelectrons and photoholes, KBrO_3_ and CH_3_OH were added separately to the Cd*_x_*Zn_1-_*_x_*S/ MR-1 systems under light as scavengers for photoelectrons and photoholes, respectively (Fig. 2d). The addition of CH_3_OH resulted in decolorization efficiencies of 93% for CdS/MR-1 and 36% for ZnS/MR-1 within 1 hour, comparable to their efficiencies in the absence of CH3OH (98% and 31%, respectively). However, with the addition of KBrO_3_, the efficiencies dropped to 72% for CdS/MR-1 and 19% for ZnS/MR-1 after 1 hour. These results demonstrated the crucial role of photoelectron transfer at the Cd*_x_*Zn_1-_*_x_*S/MR-1 interface, resulting in the enhanced system efficiency of Cd*_x_*Zn_1-_*_x_*S/MR-1 under light.

Additionally, we found that despite the higher Zn content in Cd*_x_*Zn_1-_*_x_*S/MR-1 providing a greater thermodynamic advantage for interfacial electron transfer, the apparent electron transfer efficiency decreased with increasing Zn concentration. This counterintuitive phenomenon suggests the presence of previously unidentified mechanisms that influence the preferential utilization of photoelectrons by microbes for azo dye decolorization, beyond thermodynamics considerations.

### Spectroscopic insights into interfacial ultrafast photoelectron transfer

**Fig 3:**
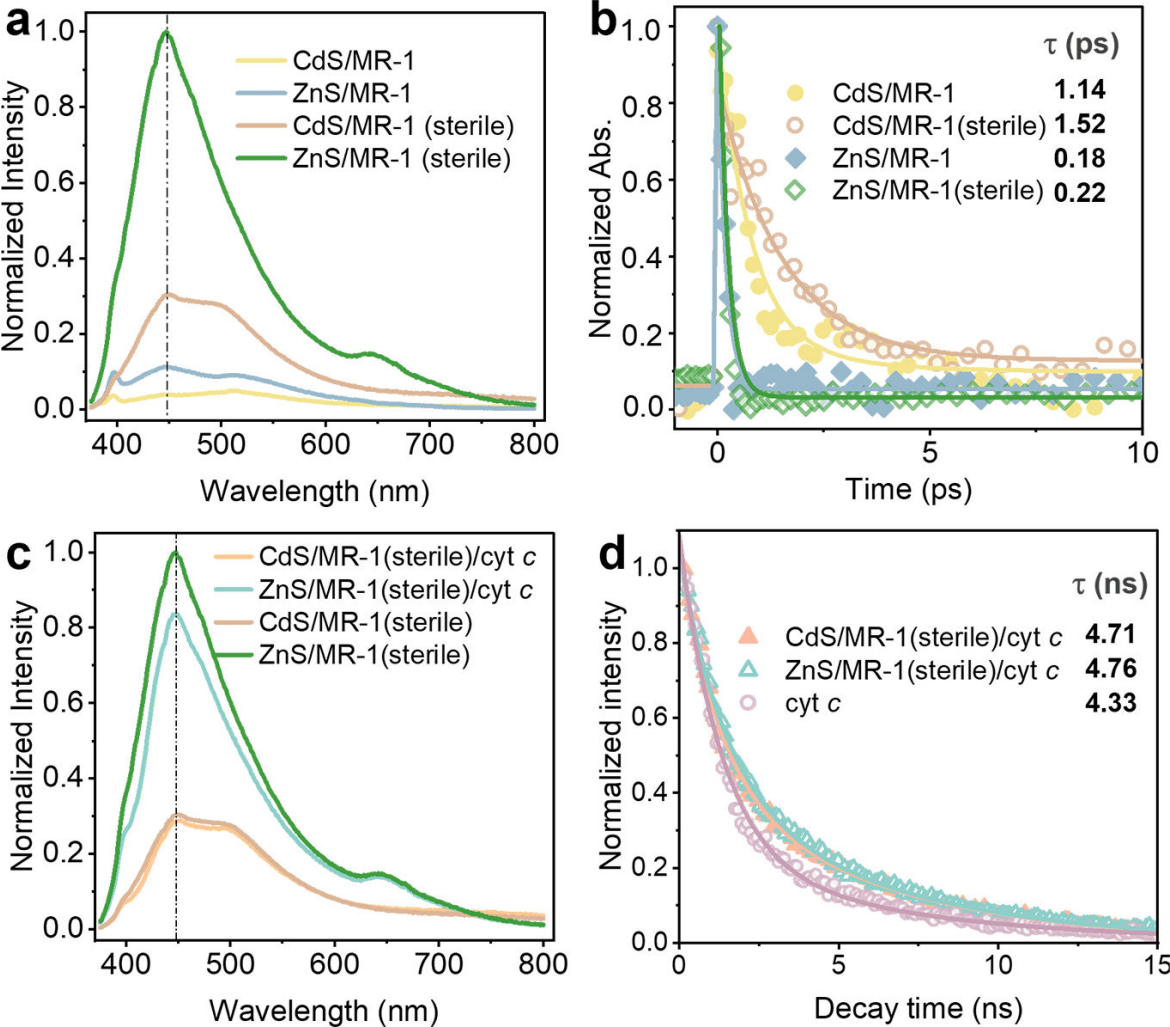
Spectroscopic measurements. (**a**) Normalized PL spectra of CdS/MR-1, ZnS/MR-1, and their sterile counterparts. (**b**) Normalized TAS of CdS/MR-1, ZnS/MR-1, CdS/MR-1 (sterile), and ZnS/MR-1 (sterile) and their sterile counterparts, with solid lines representing the kinetic fitting curves. (**c**) Normalized PL spectra of sterile CdS/MR-1 and ZnS/MR-1 with and without cyt *c*. (**d**) Normalized TRPL spectra of sterile CdS/MR-1 and ZnS/MR-1 with and without cyt *c*, with solid lines representing the kinetic fitting curves. Each experiment was repeated three times.

PL analysis was utilized to examine interfacial electron transfer processes. The PL intensity of ZnS/MR-1 and CdS/MR-1 with active MR-1 was significantly lower compared to their sterile counterparts, indicating that MR-1 serves as an effective photoelectron acceptor, thereby suppressing photoelectron-hole recombination. TAS was employed to investigate photoinduced electron transfer between Cd*_x_*Zn_1-_*_x_*S and MR-1 by monitoring the decay of excited states following pulsed light irradiation (23). Notably, ZnS systems exhibited faster decay times regardless of the presence of living MR-1, compared to CdS systems. Specifically, the average photoelectron lifetime of CdS/MR-1(sterile), CdS/MR-1, ZnS/MR-1(sterile), and ZnS/MR-1 was 1.52 ± 0.11 ps, 1.14 ± 0.12 ps, 0.22 ± 0.01 ps, and 0.18 ± 0.03 ps, respectively (Fig. 3b; Table S3). The extended photoelectron lifetime observed in the CdS systems suggests a greater likelihood of photoelectron transfer prior to recombination with photohole; thus, photoelectrons could be transferred to the intracellular electron transport chain within their lifetime. Additionally, the presence of living MR-1 slightly reduced the photoelectron lifetime in both CdS/MR-1 and ZnS/MR-1 compared to the sterile counterparts, indicating efficient photoelectron capture by MR-1 (33). Consequently, the prolonged photoelectron lifetime in CdS/MR-1 minimizes recombination and enhances interfacial photoelectron transfer kinetics, leading to increased azo dye decolorization efficiency.

In the EET chain of MR-1, external electrons are conveyed to intracellular metabolic pathways via outer-membrane (OM) cytochrome *c* (cyt *c*) proteins like OmcA and MtrC. All hybrid systems in our study exhibited distinct emission peaks at 447 nm (Fig. 3a), consistent with the PL signals of MR-1 and its extracellular OM cyt *c* (Fig. S6). To elucidate the role of cyt *c* in photoelectron transfer at the Cd*_x_*Zn_1-_*_x_*S/MR-1 interface, cyt *c* was introduced to the sterile system for spectroscopic analysis. PL measurements revealed that ZnS/MR-1(sterile)/cyt *c* and CdS/MR-1(sterile)/cyt *c* complexes displayed lower emission peaks compared to the ZnS/MR-1(sterile) and CdS/MR-1(sterile) systems (Fig. 3c), respectively, indicating that cyt *c* reduces photoelectron-hole recombination, thereby enhancing photoelectrons transfer efficiency. TRPL was conducted to further assess the carrier’s recombination rate (Fig. 3d; Table S3). The results showed that ZnS/MR-1(sterile)/cyt *c* (4.76 ± 0.04 ns) and CdS/MR-1(sterile)/cyt *c* (4.71 ± 0.01 ns) exhibited longer decay lifetimes than cyt *c* alone (4.33 ± 0.09 ns), validating more efficient spatial charge separation when cyt *c* was associated with Cd*_x_*Zn_1-_*_x_*S. However, as shown in Fig. S8, the results indicate that while cyt *c* facilitates the separation of photoelectrons, it alone is insufficient to achieve fast apparent light-driven dye decolorization. This reaction can only occur in the presence of live MR-1, where the complete Mtr photoelectron uptake pathway and dye reductases are involved. Thus, OM cyt *c* could serve as a crucial electron carrier for photogenerated charge separation and the storage of ultrafast photoelectrons at Cd*_x_*Zn_1-_*_x_*S/MR-1 interfaces. The photoelectrons from the semiconductor can be efficiently converted into valence electrons on an ultrafast timescale by OM cyt *c*, thereby linking the intracellular and EET chains in both spatial and temporal dimensions.

### Carrier mobility and band structure calculations

Spectroscopic analysis revealed that the prolonged photoelectron lifetime at the CdS/MR-1 interface enhances photoelectron transfer kinetics compared to the ZnS/MR-1 interface, despite CdS and ZnS having the same crystal structure. The results of carrier mobility calculations indicated that CdS (119.71 cm^2^/V·s) exhibited nearly twice the electron mobility of ZnS (62.47 cm^2^/V·s), while their hole mobility remained comparable (0.29 and 0.30 cm^2^/V·s, respectively) (Table 1). This increased electron mobility in CdS supports a more efficient photoelectron transfer rate at the MR-1/CdS interface.

**TABLE 1:** Degree of covalence and carrier mobilities of CdS and ZnS

Semiconductor	Degree of covalence between metal and sulfur atoms	Electron mobility in CB (cm^2^/V·s)	Hole mobility in CB (cm^2^/V·s)
CdS	82.0%	119.71	0.29
ZnS	80.6%	62.47	0.30

**Fig 4:**
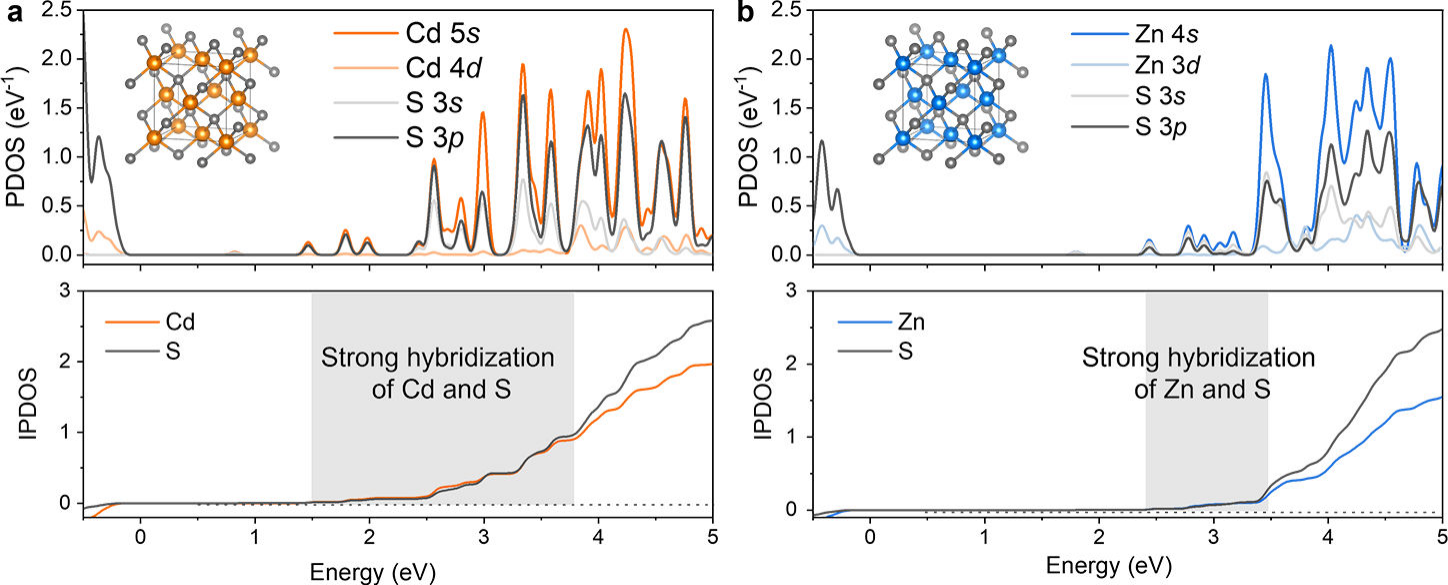
DOS at the bottom of CB. (**a**) The PDOS and IPDOS patterns of CdS. (**b**) PDOS and IPDOS patterns of ZnS. The gray shading in IPDOS patterns indicates the strong hybridization regions of metals and sulfur atoms at the bottom of CB.

Furthermore, the projected density of state (PDOS) patterns show that the majority of S 3*p* states are located in their VB for both CdS and ZnS. Notably, the bottom of the CB is primarily composed of Cd 5*s* (Zn 4*s*) states, followed by S 3*s* and 3*p* states. The remarkable overlap of metal and sulfur orbitals in the CB indicates strong hybridization, facilitating high electronic communication. The integrated PDOS (IPDOS) (Fig. 4) demonstrated that the region of significant hybridization between Cd and S at the bottom of the CB is approximately 2.2 eV, while for ZnS, it is only 1.1 eV. The wider hybridization region in CdS compared to ZnS results in more efficient electron mobility. Additionally, the degree of covalence between metal and sulfur atoms was calculated based on Pauling’s electronegativity values (Table 1). The Cd (1.69) and S (2.58) exhibit closer electronegativity values, resulting in a higher covalence degree in CdS (82.0%) compared to ZnS (80.6%), where Zn has an electronegativity of 1.65. This stronger covalency in CdS suggests a higher level of hybridization between Cd and S atoms, contributing to a faster electron transfer and extended photoelectron lifetimes.

### Transcriptomic analysis of Cd*_x_*Zn_1-_*_x_*S/MR-1 hybrid systems

**Fig 5:**
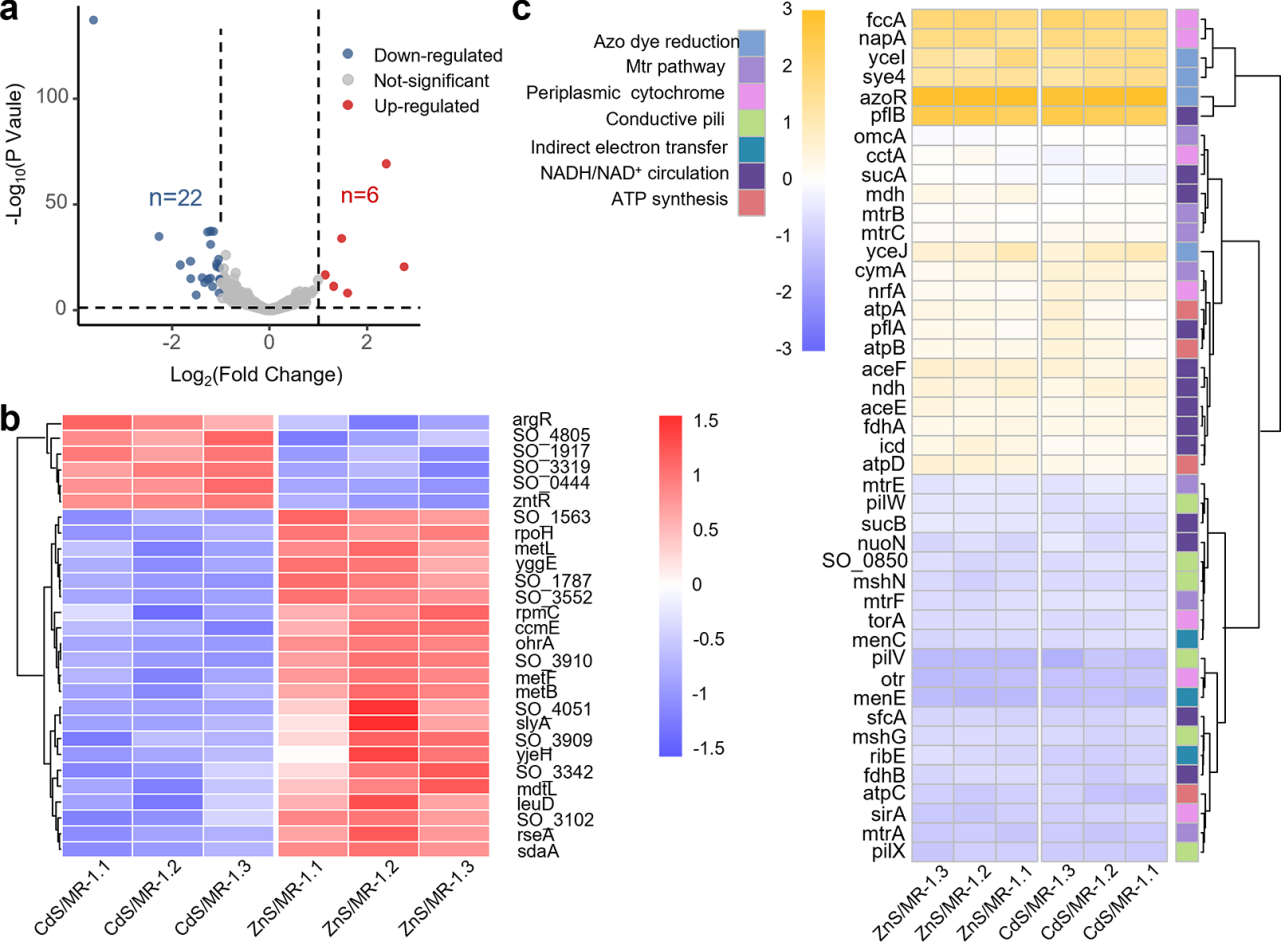
Transcriptomic analysis of CdS/MR-1 and ZnS/MR-1 after 1 hour of light irradiation during DB71 decolorization. (**a**) Volcano plots for differential gene expression analysis of CdS/MR-1 vs ZnS/MR-1. (**b**) Detailed differentially expressed genes of CdS/MR-1 vs ZnS/MR-1. (**c**) Heatmap analysis of genes involved in photoelectron uptake and utilization. CdS/MR-1.1, CdS/MR-1.2, and CdS/MR-1.3 represent three independent CdS/MR-1 groups, while ZnS/MR-1.1, ZnS/MR-1.2, and ZnS/MR-1.3 are three independent ZnS/MR-1 groups. The number of the color bar represents normalized read counts derived from RNA-seq differential expression analysis.

To elucidate MR-1’s photoelectron response to different semiconductors (CdS/ZnS), a comparative transcriptomic analysis was performed on CdS/MR-1 vs ZnS/MR-1 after 1 hour of light irradiation during DB71 decolorization. RNA-sequencing (RNA-seq) analysis revealed 4,068 gene reads mapped to the reference genome (AE014299.2). As indicated in Fig. 5a, in CdS/MR-1, six genes were upregulated and 22 genes were downregulated compared to ZnS/MR-1 (*P* < 0.05, |log_2_FoldChange| > 1), with less than 1% showing more than a twofold change in expression. Detailed differentially expressed genes are presented in Fig. 5b. Functions of these genes were annotated using Gene Ontology (GO) terms and Kyoto Encyclopedia of Genes and Genomes (KEGG) pathways in Fig. S9. The analysis revealed that differentially expressed genes were involved in various biological processes, including cellular processes, metabolic processes, response to stimuli, biological regulation, localization, and detoxification. Changes in gene expression affected molecular functions such as binding, catalytic activity, transporter activity, transcription regulator activity, and structural molecule activity. In addition, differentially expressed genes impacted cellular components, especially protein-containing complexes. KEGG pathway analysis indicated significant overexpression of genes related to amino acid metabolism in ZnS/MR-1.

**Fig 6:**
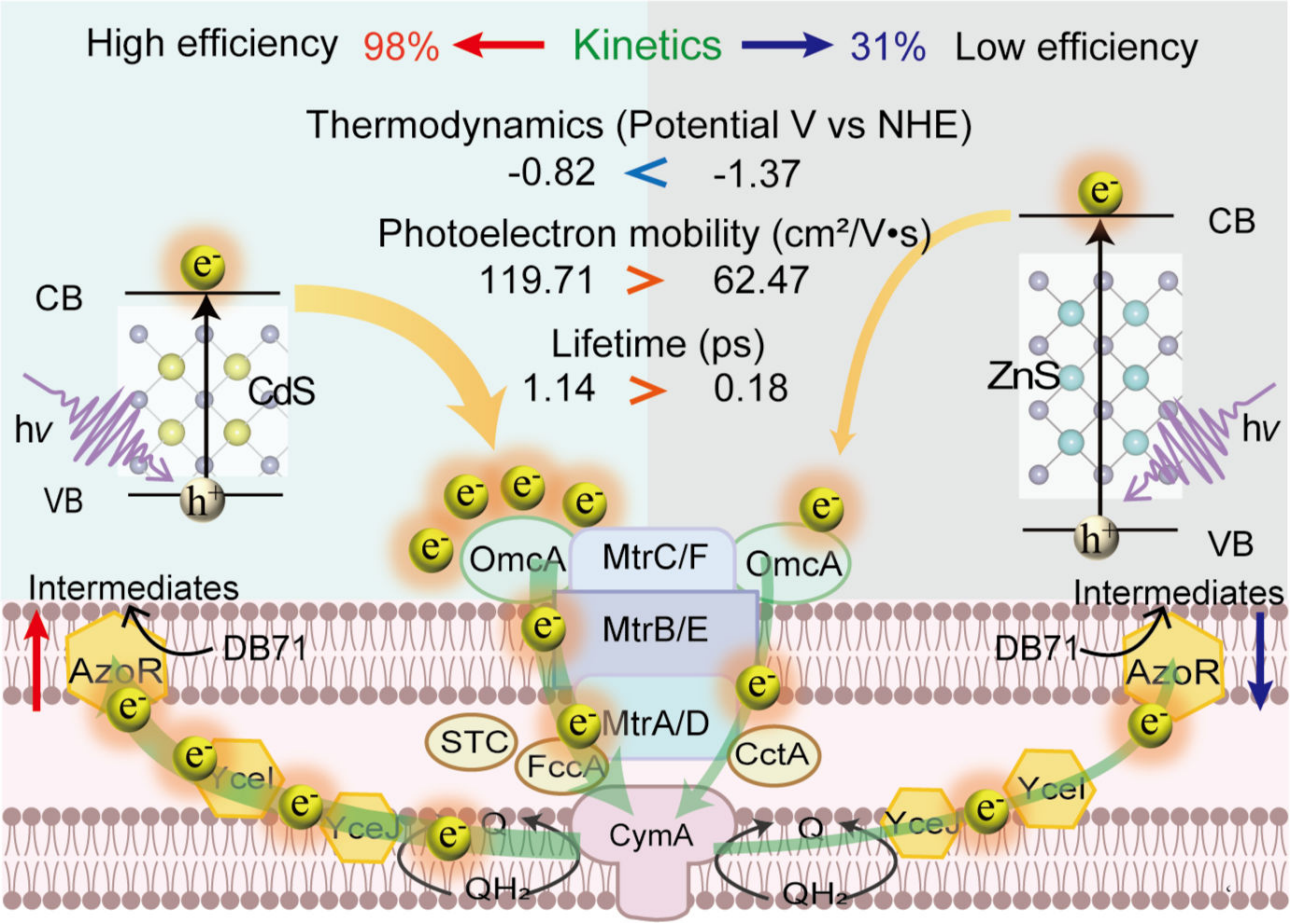
Schematic illustration of the ultrafast electron transfer mechanism at Cd*_x_*Zn_1-_*_x_*S/MR-1 interface.

Specifically, zntR and SO_0444 are involved in the response to Cd^2+^ and Zn^2+^. To investigate, we measured the concentration of Cd^2+^ and Zn^2+^ in the solution after 5 hours of the decolorization reaction. As shown in Fig. S7, less than 0.02 mM Cd^2+^ and Zn^2+^ leached out. We then conducted a CCK-8 assay to assess the impact of 0.02 mM of Cd^2+^ and Zn^2+^ on cell viability and found no significant toxicity. Therefore, although zntR and SO_0444 were upregulated in CdS/MR-1, their expression appears to be a response to specific metal ions rather than a major factor influencing the decolorization process. Meanwhile, these differentially expressed genes are not involved in the reported pathway for potential photoelectron transfer (including the Mtr pathway with OmcA, MtrC, MtrA, MtrB, MtrF, CymA, etc.; conductive pili with mshG, mshN, pilX, pilW, etc.; and indirect electron transfer with menC, menE, ribE, etc.) and utilization (azo dye reduction pathway with yceI, yceJ, sye4, azo dye reductase (azoR), etc.; NADH/NAD^+^ cycling with aceE, aceF, pflB, pflA, nuoN, ndh, etc.; and ATP synthesis with atpA, atpB, etc.) (34–36). As shown in Fig. 5c, this indicated that different photoelectrons from CdS and ZnS did not cause notable gene expression changes in the reported photoelectron uptake and utilization. Among the genes, those related to the Mtr pathway exhibited higher expression levels compared to those involved in indirect electron transfer and conductive pili, underscoring the Mtr pathway’s importance in photoelectron uptake. The differential gene expression analysis suggested that MR-1 employed similar electron transfer pathways for capturing photoelectrons from CdS or ZnS. As illustrated in Fig. 6, the proposed possible photoelectron utilization pathway involves photoelectron transfer through OM cytochromes (e.g., OmcA and MtrC), outer porin (e.g., MtrB), periplasmic cytochromes (e.g., FccA), and inner membrane-associated quinol oxidase (CymA) to the azoR.

### Mechanism of ultrafast electron transfer for enhanced microbial photoelectron utilization

Based on our findings, an ultrafast electron transfer mechanism at the Cd*_x_*Zn_1-_*_x_*S/MR-1 interface for efficient azo dye reduction is proposed in Fig. 6. In this study, Cd*_x_*Zn_1-_*_x_*S nanoparticles were *in situ* synthesized on the MR-1 surface, achieving a close spatial contact. All Cd*_x_*Zn_1-_*_x_*S compounds possess thermodynamically favorable energy levels for photoelectron transfer to MR-1, with CdS (−0.82 V vs NHE) being less thermodynamically favorable than ZnS (−1.37 V vs NHE) (Fig. 1d). However, the decolorization efficiency of DB71 by CdS/MR-1 (98%) was three times higher than that by ZnS/MR-1 (31%) within the same reaction time (Fig. 2b). This suggests that CdS provides more available photoelectron per unit time, resulting in more efficient electron transfer at the CdS/MR-1 interface. The longer photoelectron lifetime and faster photoelectron mobility in CdS notably enhance photoelectron transfer kinetics to MR-1 before recombination with photohole (Fig. 3; Table S3). Spectroscopic changes observed in TAS (Fig. 3b), PL (Fig. 3c), and TRPL (Fig. 3d) indicate that photoelectrons are effectively captured with the assistance of OM cyt *c* in MR-1. These captured photoelectrons are then transferred to azoR, facilitating the decolorization of DB71 (Fig. 5c). Despite differences in extracellular and intracellular electron transfer kinetics, OM cyt *c* in MR-1 can capture photoelectrons within their lifetime. Once captured, these photoelectrons are stored in steady-state reduced cyt *c*, which has a long lifetime compared to the transient excited-state photoelectrons. This process modulates extracellular and intracellular electron transfer kinetics at the semiconductor/microbe interface. Given the similar electron transfer pathways in CdS/MR-1 and ZnS/MR-1, the higher accumulation of photoelectrons per unit time at the CdS/MR-1 interface ensures a greater concentration of available photoelectrons, enhancing DB71 decolorization efficiency. Consequently, the kinetic advantage conferred by the extended photoelectron lifetime translates into a thermodynamic advantage in electron transfer, highlighting the importance of photoelectron transfer kinetics in optimizing the performance of semiconductor-microbe hybrid systems and broadening their applications in solar-driven environmental remediation.

### Environmental implications

These findings not only offer valuable insights into optimizing the design of more efficient semiconductor-microbe hybrid systems but also pave the way for the development of scalable and highly effective environmental remediation strategies. Moreover, in solar-related natural matrices where light-sensitive semiconductor elements (e.g., natural minerals) and microbes coexist, mineral photoelectrons may influence microbial diversity and functionality, thereby more broadly impacting natural ecosystems (11, 37, 38). Understanding the ultrafast kinetics of mineral photoelectron utilization by non-photosynthetic microbes improves our comprehension of the environmental significance of light in biogeochemical processes and offers new theoretical foundations for microbial energy metabolism.

### Conclusions

This study confirms that non-photosynthetic microbe, similar to photosynthetic microbe, can efficiently utilize photoelectron energy through ultrafast electron transfer pathways, not only in CdS/MR-1 system but also in a series of Cd*_x_*Zn_1-_*_x_*S/MR-1 systems. By further investigating the ultrafast electron transfer kinetics at the Cd*_x_*Zn_1-_*_x_*S/MR-1 interface, we found that, despite ZnS’s greater thermodynamic potential, CdS’s longer photoelectron lifetime and higher electron mobility provide kinetic advantages that enhance the non-photosynthetic microbial utilization of photoelectrons. Additionally, the OM cyt *c* could play a pivotal role in capturing and transferring photoelectrons within their lifetime, modulating electron transfer kinetics at the semiconductor/microbe interface. While the energy of photoelectrons and interfacial photoelectron transfer kinetics do not alter the photoelectron uptake pathways, the kinetic advantages at the CdS/MR-1 interface lead to greater accumulation of bioavailable photoelectrons per unit time, thereby benefiting microbe activity and enhancing system efficiency.

Future research in this area can focus on two main directions. First, investigating the impact of environmental factors such as pH, temperature, and organic contaminants on photoelectron transfer kinetics will help optimize semiconductor/microbe system performance under varying natural conditions. Second, further exploration of the interactions between semiconductor and microbial components, including extracellular enzymes and electron mediators, will deepen our understanding of microbial photoelectron energy utilization.

## MATERIALS AND METHODS

### Bacterial culture and construction of Cd*_x_*Zn_1-_*_x_*S/MR-1 hybrid systems

*S. oneidensis* MR-1 used in this study was obtained from China Center for Type Culture Collection and cultivated aerobically in Luria-Bertani (LB) medium (10.0 g L^−1^ peptone, 5.0 g L^−1^ yeast extract, and 10.0 g L^−1^ NaCl, pH 7.0) overnight at 35°C and 140 rpm. Then, the cultured MR-1 was collected by centrifugation (5,000 rpm, 10 min) and resuspended to an OD_600_≈0.35 in 50 mM HEPES, 50 mM NaCl, 10 mM lactate, and 1% LB, pH 7.0. For the construction of Cd*_x_*Zn_1-_*_x_*S hybrid systems, 1 mM cysteine was added into MR-1 suspensions. This was followed by the addition of CdCl_2_ and ZnSO_4_ in varying proportions, maintaining their total concentration at 0.3 mM. After 48 h incubation at 30°C and 150 rpm in a shake incubator, the Cd*_x_*Zn_1-_*_x_*S/MR-1 hybrid system was constructed and collected by centrifugation (5,000 rpm, 10 min) (as detailed in Fig. S1).

### Cell viability and TEM of Cd*_x_*Zn_1-_*_x_*S/MR-1 hybrid systems

The cell viability in Cd*_x_*Zn_1-_*_x_*S was assessed using a CCK-8 assay kit (obtained from Beijing Solarbio Science & Technology Co., Ltd). Following the standard protocol of the CCK-8 assay, the optical density at 450 nm of each sample was measured by microplate reader (800 TS, BioTek, USA). Cell viability was determined using Eq. 1:


 (Eq. 1) $$cell\;viability\;\left(\%\right)=\frac{\left(A_{experimental}-A_{blank\;}\right)}{\left(A_{control}-A_{blank\;}\right)}\times 100\%.$$

where *A*_experimental_ refers to the absorbance of the experimental group with Cd*_x_*Zn_1-_*_x_*S and MR-1, *A*_control_ is the absorbance of the control group (only MR-1), and *A*_blank_ is the absorbance of blank group (only cell culture medium).

We further used the LIVE/DEAD Bacterial Viability Kit (obtained from Beyotime Biotechnology) with DMAO and PI to assess the percentage of live bacteria. DMAO exhibits green fluorescence with *E*_*x*_/*E*_*m*_ = 503/530 nm, indicating live bacteria, while PI exhibits red fluorescence with *E*_*x*_/*E*_*m*_ of 535/617 nm, representing dead bacteria. Fluorescence values (FU) were detected using a fluorescence microplate reader (BioTek Cytation5). The percentage of live bacteria was determined using Eq.2:


 (Eq. 2) $$Percentage\;of\;live\;bacteria=\frac{FU_{green}}{\left(FU_{green}+FU_{red\;}\right)}\times 100\%.$$

TEM was used to observe the distribution of biosynthesized Cd*_x_*Zn_1-_*_x_*S in MR-1 cells. The samples for TEM were fixed in 2.5% glutaraldehyde overnight and stained with 1% osmium tetroxide for 1 h, dehydrated in graded series of ethanol (50%, 70%, 80%, 90%, and 100% with 15 min for each level). The samples were then embedded in Eponate resin, sliced into pieces by an ultramicrotome (EM UC7, Leica, Germany), and observed through TEM (JEM-F200, JEOL, Japan).

### Characterization of Cd*_x_*Zn_1-_*_x_*S/MR-1 hybrid systems

The Cd*_x_*Zn_1-_*_x_*S/MR-1 samples underwent three washes with deionized water followed by freeze-drying for further characterization. The phase composition of Cd*_x_*Zn_1-_*_x_*S was characterized using an X-ray diffractometer (X’pert pro, PANalytical, Netherlands). UV-vis diffusive reflectance spectra were obtained using a UH4150 spectrophotometer (Hitachi, Japan), and the band gaps were calculated via Tauc plots. Surface chemical states and the VB edge of Cd*_x_*Zn_1-_*_x_*S samples were analyzed through XPS on an ESCALAB250 spectrometer (Thermo Fisher Inc., USA). The concentration of Cd^2+^ and Zn^2+^ in Cd*_x_*Zn_1-_*_x_*S solid was measured using an Inductively Coupled Plasma-Atomic Emission Spectrometer (ICP-OES, Prodigy 7, Teledyne Leeman Labs, USA) following sample digestion with concentrated hydrochloric acid.

### Decolorization experiments

To prepare for the decolorization experiments, the Cd*_x_*Zn_1-_*_x_*S/MR-1 composites were washed twice with 50 mM phosphate buffered saline (PBS) (11.55 g L^−1^ Na_2_HPO_4_·12H_2_O, 2.77 g L^−1^ NaH_2_PO_4_·2H_2_O, 0.13 g L^−1^ KCl, and 0.31 g L^−1^ NH_4_Cl, pH = 7.00). The washed composites were then resuspended in 50 mM PBS supplemented with 10 mM lactate. These suspensions were transferred to anaerobic quartz bottles, each containing 100 mg/L of DB71, and exposed to wideband UV light (280−400 nm) with an intensity of 1.05 mW/cm² to initiate the decolorization process. Samples were periodically collected using a syringe, centrifuged, and analyzed for DB71 concentration using an Evolution 220 spectrophotometer (Thermo Fisher Scientific, USA) at 583 nm. The DB71 decolorization efficiency and decolorization rate were defined as Eq. 3 and Eq. 4:


(Eq. 3) $$Decolorization\;efficiency=\frac{C_{t}}{C_{o}}$$

where *C*_*t*_ is the concentration of the dye at time *t* and *C*_*0*_ is the initial concentration of the dye.


(Eq. 4) $$Decolorization\;rate=\left(1-\frac{C_{t}}{C_{o}}\right)\times 100\%$$

representing the percentage of dye decolorized over time.

To clarify the contributions of MR-1 and Cd*_x_*Zn_1-_*_x_*S to DB71 decolorization, control experiments include DB71 without Cd*_x_*Zn_1-_*_x_*S/MR-1, DB71 with solely MR-1, and DB71 with sterilized Cd*_x_*Zn_1-_*_x_*S/MR-1 (121°C for 20 min, denoted as Cd*_x_*Zn_1-_*_x_*S/MR-1 [sterile]). DB71 with Cd*_x_*Zn_1-_*_x_*S/MR-1 in the presence of 0.5 mM KBrO_3_ or 0.5 mM CH_3_OH was conducted to confirm the roles of photoelectron and photohole. All reagents used were of analytical grade, and each experimental condition was performed in triplicate.

### Spectroscopic measurements

PL and TRPL measurements were conducted at room temperature using a fluorescence spectrophotometer (FLS-1000, Edinburgh Instruments, UK). For PL spectra, samples were excited at 350 nm, with emission detected in the 375–800 nm range. TRPL spectra were obtained with an excitation wavelength of 375 nm and detection at 450 nm, corresponding to the peak emission identified in the PL spectra.

TA spectroscopy measurements were performed using a regenerative-amplified Ti:sapphire laser (Coherent, Legend Elite) with 25-fs pulses at 1 kHz, coupled with a Helios pump-probe system (Ultrafast Systems). The excitation wavelength for TAS was set at 320 nm, and detection was carried out at 450 nm, based on prior studies (23).

To quantitatively analyze the electron transfer kinetics, we performed kinetic analysis with exponential fittings for TAS data and TRPL data. The kinetics were described by a biexponential function, as defined in Eq. 5 (24)


(Eq. 5) $$N\left(t\right)=N_{1}e^{-t/\tau 1}+\;N_{2}e^{-t/\tau 2}.$$

The average lifetime *τ* of the sample was calculated by Eq. 6:


(Eq. 6) $$\tau =(N_{1}\tau_{1}^{2}+\;N_{2}\tau_{2}^{2})\;/\;(N_{1}\tau_{1}+\;N_{2}\tau_{2}).$$

To elucidate the role of cyt *c* in photoelectron transfer at the Cd*_x_*Zn_1-_*_x_*S/MR-1 interface, cyt *c*, which is commercially sourced from Coolaber (China), was introduced into the sterile system at a concentration of 0.5 g/L for both spectroscopic analysis and decolorization experiments.

### Transcriptomic analysis of Cd*_x_*Zn_1-_*_x_*S/MR-1

For transcriptome analysis, samples from light irradiation group at 1 hour of DB71 decolorization were centrifuged to remove medium, and the cells were immediately frozen in liquid nitrogen. These frozen samples were then sent to Guangdong MAGIGENE Technology Co., Ltd. (MAGIGENE, Guangzhou, China) for RNA extraction and subsequent transcriptome analysis. All experiments were performed in triplicate. The RNA-seq results were aligned to the *S. oneidensis* MR-1 complete genome (AE014299.2) available on the National Center for Biotechnology Information, which refers to chromosomal genome. The differential genes were annotated using the KEGG pathway and categorized according to GO terms into three domains: biological process, cellular component, and molecular function. The original data of heatmaps are normalized read counts derived from RNA-Seq differential expression analysis. These counts were calculated using the R packages (library [dplyr], library [tidyr], library [tximport], and library [DESeq2]). The heatmaps plot was generated using R software (v.4.2.2) with the “pheatmap” package (v.1.0.12) through Hiplot Pro (https://hiplot.com.cn/) (39).

## Data Availability

All data generated during this study are included in the published article and its supplemental material.

## SUPPLEMENTAL MATERIAL

Supplemental material aem.00138-25-s0001.docx Fig. S1 to S9, Tables S1 to S3, and details of carrier mobility and band structure calculations for CdxZn1-xS.

ASM does not own the copyrights to Supplemental Material that may be linked to, or accessed through, an article. The authors have granted ASM a non-exclusive, world-wide license to publish the Supplemental Material files. Please contact the corresponding author directly for reuse.
