# Supplementary material for: Ultrafast electron transfer kinetics at semiconductor-microbe interface: key to efficient extracellular photoelectron utilization
Source: Appl Environ Microbiol. 2025 Jul 31;91(8):e00138-25. doi: 10.1128/aem.00138-25 (PMC12366296; doi:10.1128/aem.00138-25)
Supplement: Supplemental material — Fig. S1 to S9, Tables S1 to S3, and details of carrier mobility and band structure calculations for CdxZn1-xS. [file aem.00138-25-s0001.docx]

**Supplementary materials**

**Ultrafast Electron Transfer Kinetics at Semiconductor-Microbe Interface: Key to Efficient Extracellular Photoelectron Utilization**

Yimei Du ^a,b^, Yan Li ^a,b*^, Yanzhang Li ^a,b^, Song Jin ^c^, Huan Ye ^a,b^, Bingxu Hou ^a,b^, Tianci Hua ^a,b^, Jiaqi Zhu ^a,b^, Houze Lu ^a,b^, Anhuai Lu ^a,b^, Tao Li ^d^

^a^ SKLab-DeepMinE, MOEKLab-OBCE, School of Earth and Space Sciences, Peking University, Beijing 100871, China.

^b^ Beijing Key Laboratory of Mineral Environmental Function, School of Earth and Space Sciences, Peking University, Beijing 100871, China.

^c^ Department of Civil and Architectural Engineering, University of Wyoming, Laramie, WY 82071, USA.

^d^ Key Laboratory of Algal Biology, Institute of Hydrobiology, Chinese Academy of Sciences, Wuhan 430072, China.

* Author for correspondence (E-mail: liyan-pku@pku.edu.cn)

**Materials and Methods**

**Calculation of carrier mobility and band structure of Cd*_x_*Zn_1-_*_x_*S****.** Carrier mobility of CdS and ZnS were calculated based on deformation potential (DP) theory.^1^ DFT-based first-principles calculations were performed to get the electronic structure, band structure, and elastic constant by means of the projected augmented wave method using Vienna Ab initio Simulation Package (VASP).^2-4^ The generalized gradient approximations with the Perdew−Burke−Ernzerhof (PBE) version were adopted to treat the exchange and correlation (XC) functional.^5^ We employed pseudopotentials to model the ion-electron interaction. The plane wave energy cutoff was set to 800 eV and the energy convergence criterion for the electronic self-consistent calculations was 10^-8^ eV. The force difference was converged to 1×10^-3^ eV/Å. The k-points grids were set as 5 × 5 × 5 and 10 × 10 × 10 in the Brillouin zone in structural relaxation calculations and electronic structure calculations using the Monkhorst-Pack method.^6^

Band struction calculations were performed using the VASP. The sampling of the Brillouin zone was performed using a Gamma scheme. The applied k-mesh for the structure optimization was set corresponding to k spacing of 0.3 Å^-1^. The PAW potentials were chosen Cd 4*d*^10^5*s*^2^, Zn 3*d*^10^4*s*^2^, and S 3*s*^2^3*p*^4^ as valence electrons from the VASP database. The force convergence criterion was set as 0.01 eV Å^-1^ when the lattice parameters were relaxed. Atomic positions were relaxed and the criterion for energy convergence was taken to be 0.01 meV. The cutoff for the plane-wave was chosen to be 500 eV. For primitive cell, the Gamma-centered k-point grid was set as 3 × 3 × 3 and 7 × 6 × 7 for geometry optimization and energy calculations, respectively. While 7 × 6 × 7 and 14 × 12 × 13 of k-point meshes were applied for supercell respectively. The generation of input files and analysis of electron structure was used vaspkit code.^7^

**
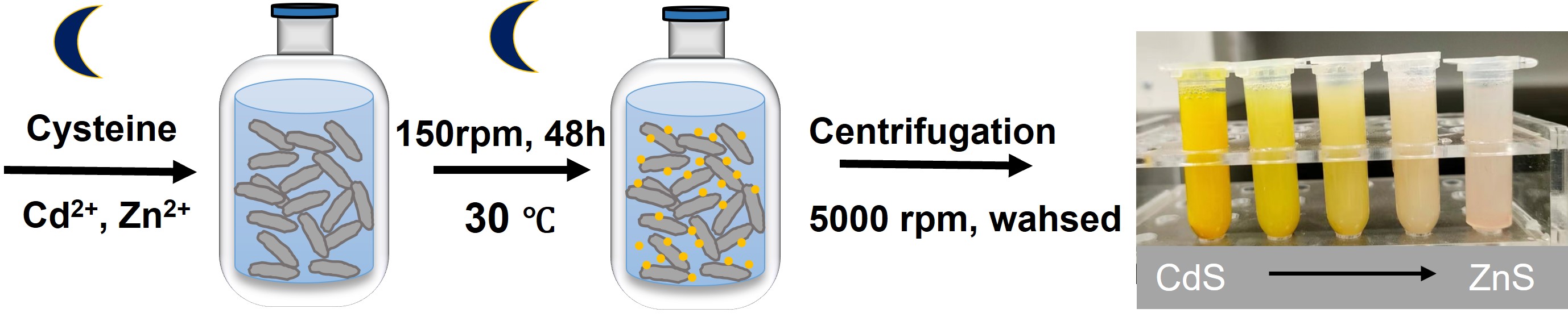
**

**Fig. S1** Schematic diagram of Cd*_x_*Zn_1-_*_x_*S/MR-1 hybrid systems construction. (the right is the actual picture of Cd*_x_*Zn_1-_*_x_*S/MR-1 hybrid systems)


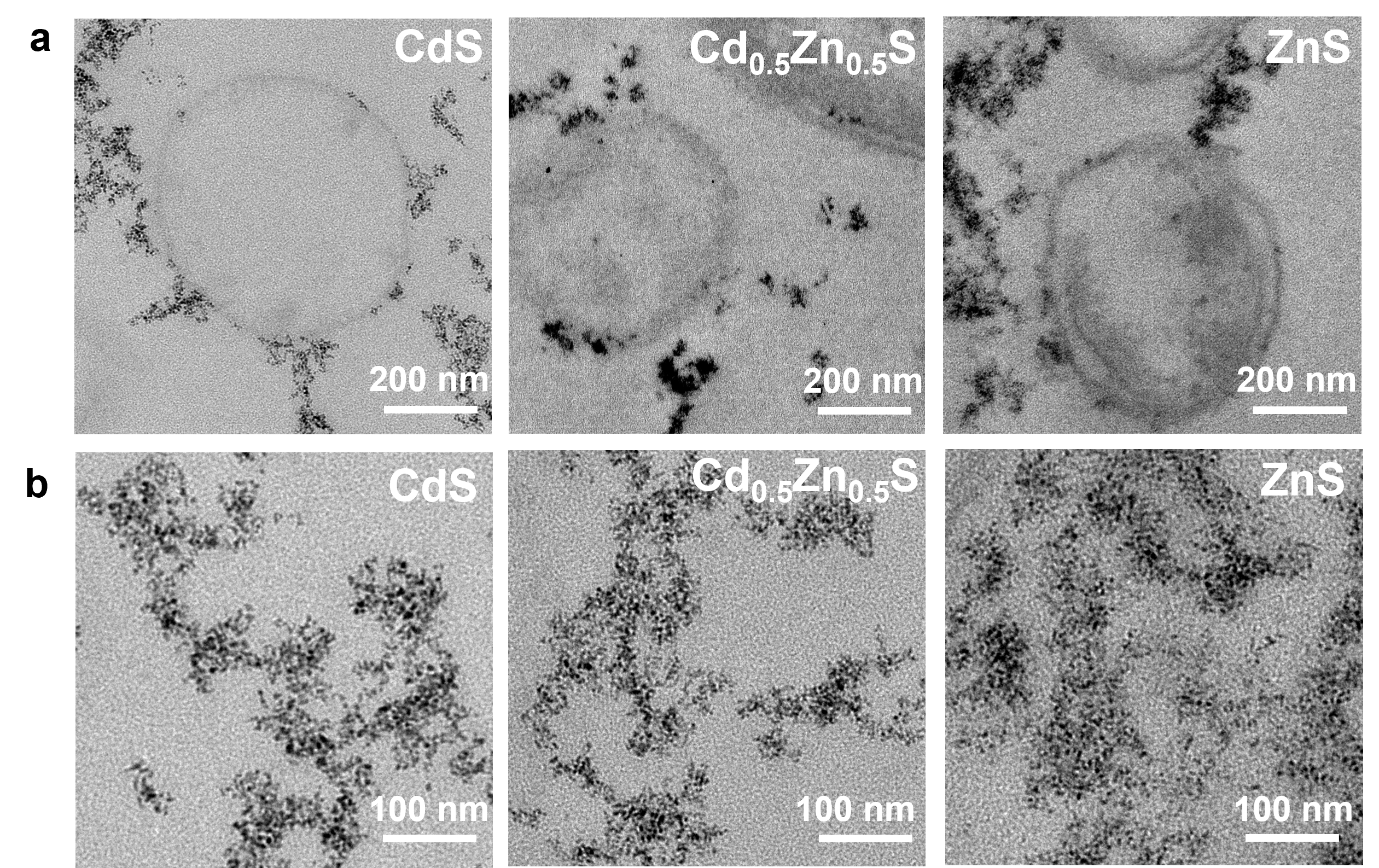


**Fig. S2** TEM images (a) TEM image of Cd*_x_*Zn_1-_*_x_*S/MR-1 interface, where Cd*_x_*Zn_1-_*_x_*S nanoparticles are in combination with MR-1 cell. (b) TEM image of Cd*_x_*Zn_1-_*_x_*S nanoparticles, cropped using GMS (Gatan Microscopy Suite) software.

.


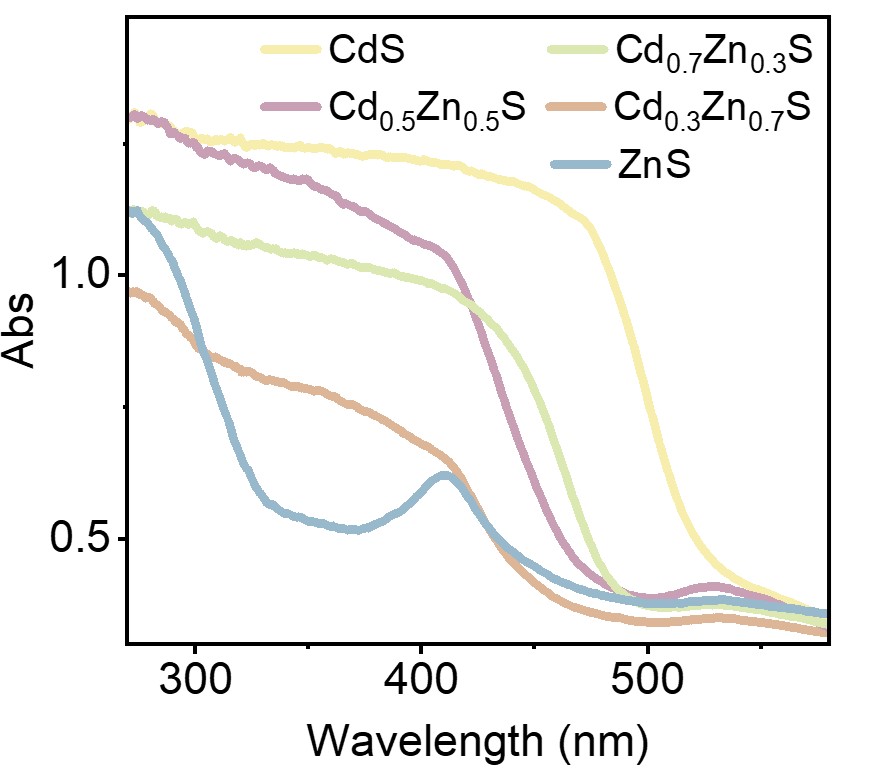


**Fig. S3** UV-vis spectra of Cd*_x_*Zn_1-_*_x_*S/MR-1.


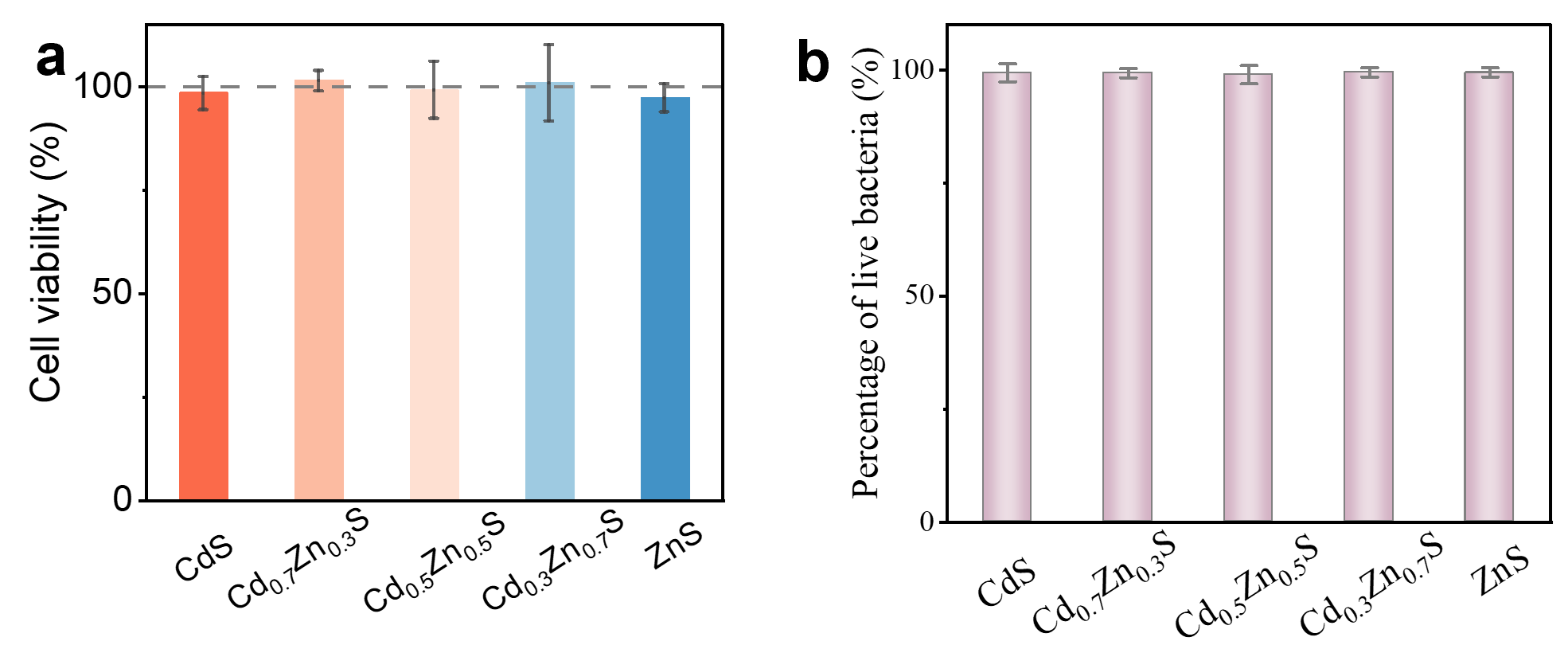


Fig. S4 Cell viability of Cd*_x_*Zn_1-_*_x_*S/MR-1 hybrid systems. (a) CCK8 test of Cd*_x_*Zn_1-_*_x_*S/MR-1. (b) LIVE/DEAD Bacterial Viability test of Cd*_x_*Zn_1-_*_x_*S/MR-1.


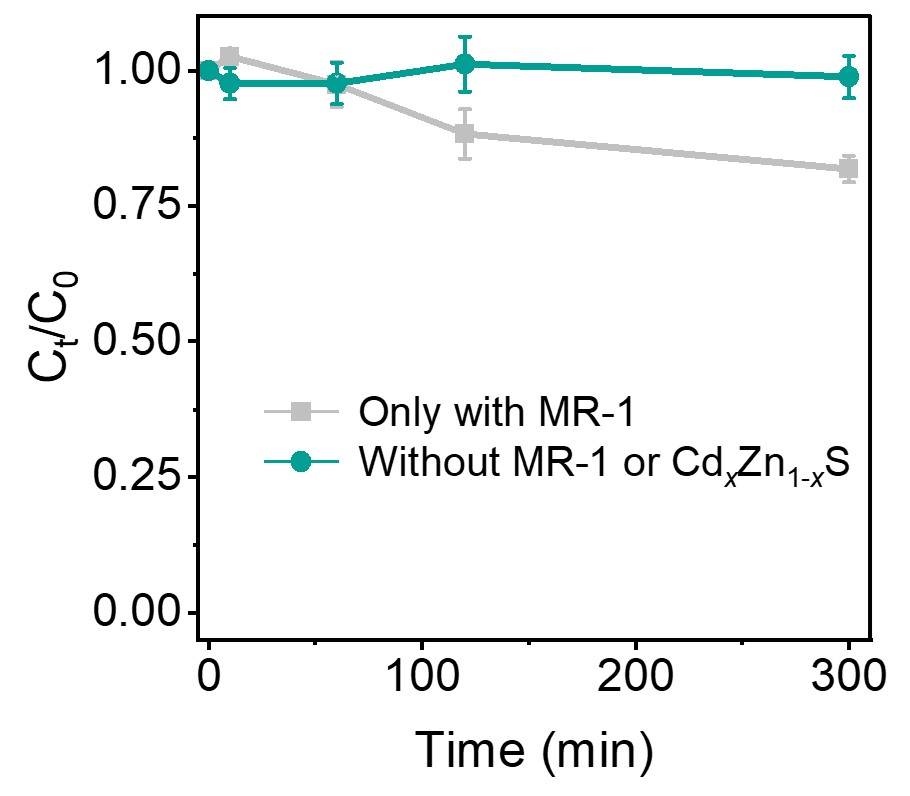


**Fig. S5** Decolorization of DB71 with only MR-1 or without any additives (MR-1 or Cd*_x_*Zn_1-_*_x_*S).

**Fig. S6** PL spectra of MR-1 and cyt *c* excited by 350 nm.


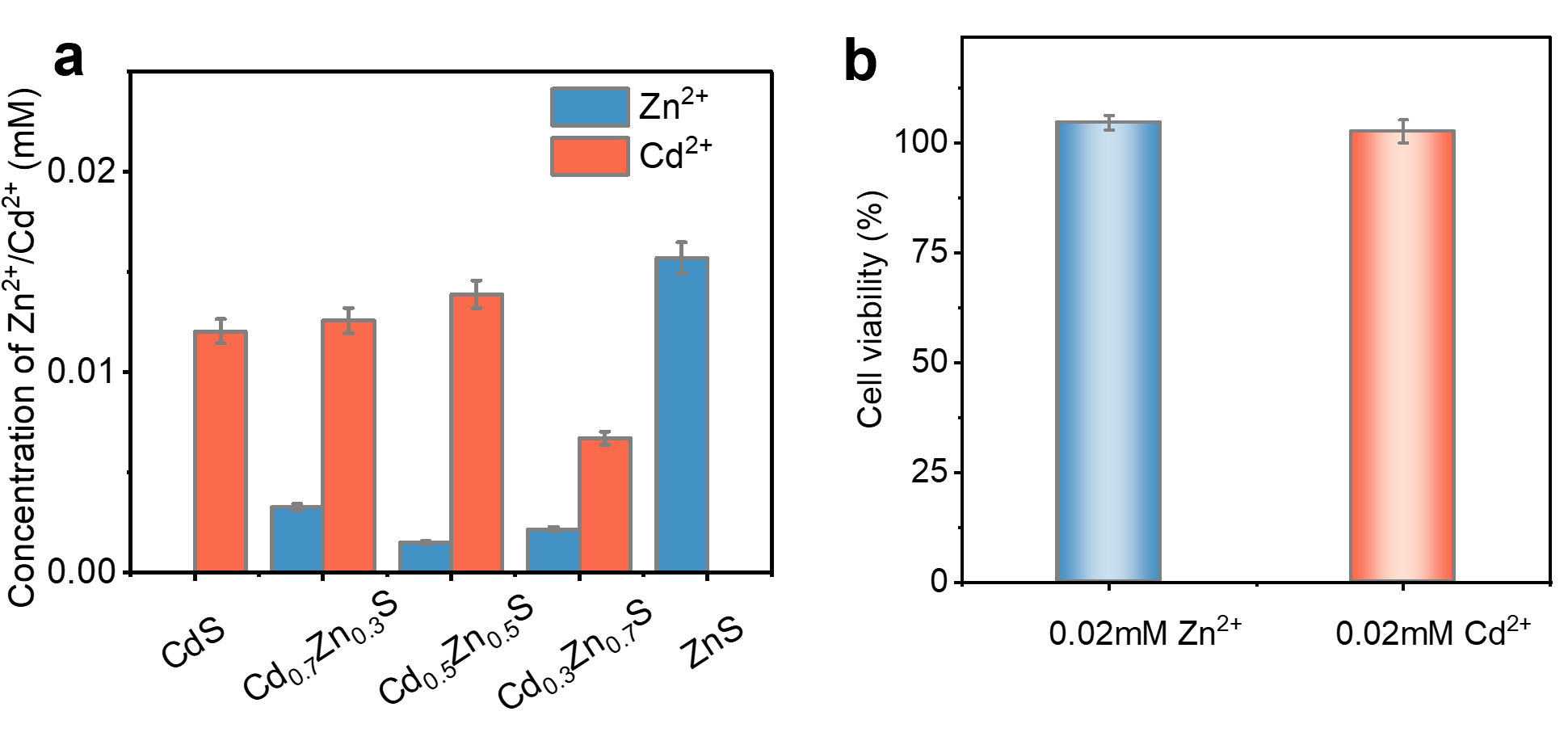


**Fig. S7** Toxicity assessment of Cd²⁺ and Zn²⁺ leaching after the decolorization reaction. (a) The concentration of Cd²⁺ and Zn²⁺ after the decolorization reaction. (b) CCK-8 test of 0.02 mM Cd²⁺ and Zn²⁺ on MR-1 cells.


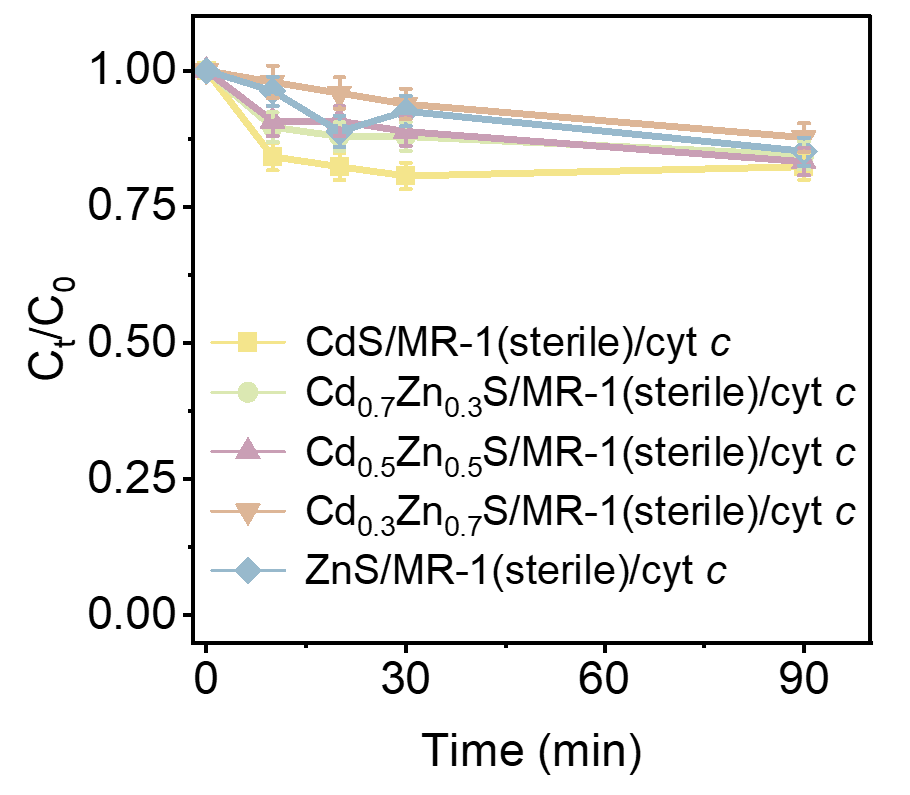


**Fig. S8** Decolorization curves of DB71 with increasing irradiation time with cyt *c*. Each experiment has been repeated three times to calculate error bars.


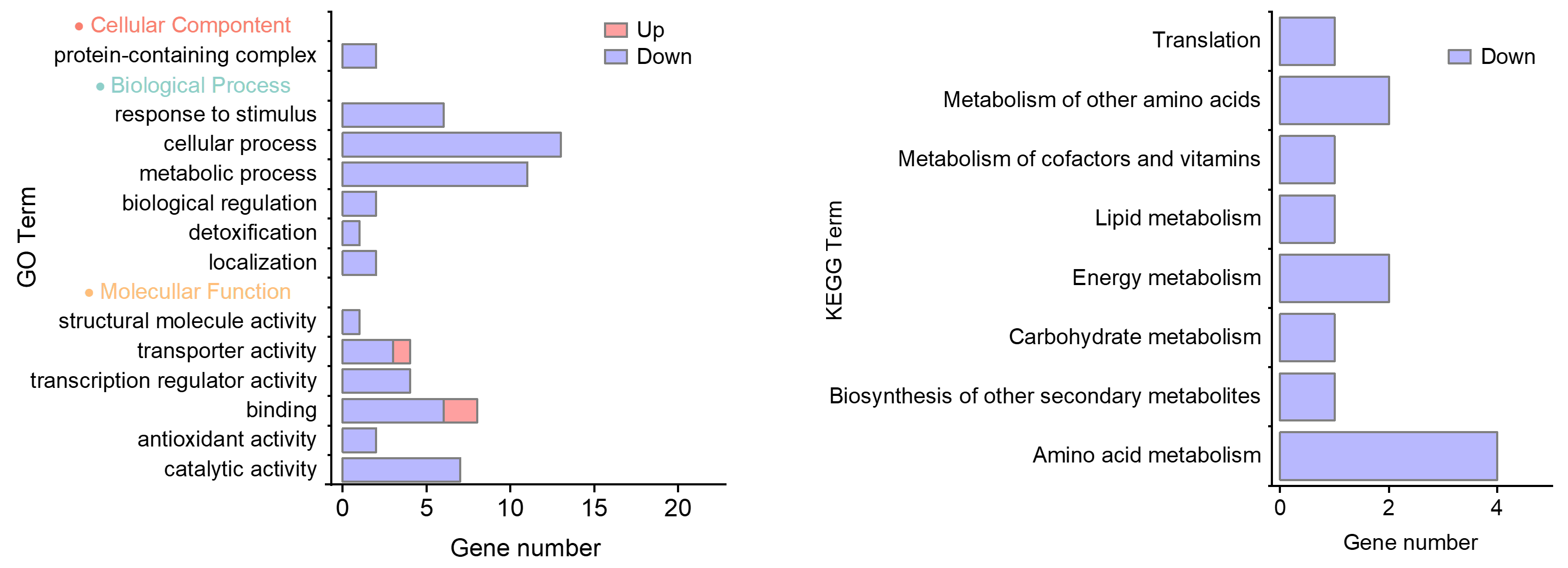


**Fig. S9** GO Function and KEGG Pathway classification of different genes in CdS/MR-1 vs ZnS/MR-1 after 1h light irradiation.

**Table S1.** Concentration of Zn^2+^ and Cd^2+^ of Cd*_x_*Zn_1-_*_x_*S/MR-1

| **Zn^2+^ (mM)** | **Cd^2+^ (mM)** | **Molar ration of Zn^2+^/Cd^2+^** | Cd*_x_*Zn_1-_*_x_*S |
| --- | --- | --- | --- |
| 0 | 0.58±0.010 | 0 | CdS |
| 0.13±0.003 | 0.26±0.005 | 0.5 | Cd_0.7_Zn_0.3_S |
| 0.26±0.009 | 0.26±0.010 | 1.0 | Cd_0.5_Zn_0.5_S |
| 0.27±0.001 | 0.11±0.002 | 2.5 | Cd_0.3_Zn_0.7_S |
| 0.42±0.004 | 0 | Not available | ZnS |

**Table S2.** Energy level and potentials with respect to the normal hydrogen electrode (NHE) for VB and CB of Cd*_x_*Zn_1-_*_x_*S/MR-1 at pH=7.0

| **Samples** | **E_CB_ (eV vs. AVS)** | **E_CB_ (V vs. NHE)** | **E_VB_ (V vs. NHE)** |
| --- | --- | --- | --- |
| CdS | -3.98 | -0.82 | 1.55 |
| Cd_0.7_Zn_0.3_S | Not available | -0.87 | 1.68 |
| Cd_0.5_Zn_0.5_S | Not available | -1.00 | 1.68 |
| Cd_0.3_Zn_0.7_S | Not available | -1.08 | 1.68 |
| ZnS | -3.46 | -1.37 | 2.28 |

**Table S3.** Biexponential fitting data for TAS and TRPL spectra

|  | **Sample** | | **τ_1_ (ps)** | **τ_2_ (ps)** | **R^2^** | **τ (ps)** |
| --- | --- | --- | --- | --- | --- | --- |
| **TAS** | CdS/MR-1 | 0.7616±0.0593 | | 2.4±0.2563 | 0.9446 | 1.14±0.12 |
|  | ZnS/MR-1 | 0.1886±0.0306 | | 0.0011±0.0002 | 0.9526 | 0.18±0.03 |
|  | CdS/MR-1(sterile) | 1.5157±0.1454 | | 1.5157±0.0761 | 0.9490 | 1.52±0.11 |
|  | ZnS/MR-1(sterile) | 0.2177±0.0112 | | 0.2177±0.0117 | 0.9584 | 0.22±0.01 |
|  | **Sample** | **τ_1_ (ns)** | | **τ_2_ (ns)** | **R^2^** | **τ (ns)** |
| **TRPL** | CdS/MR-1(sterile)/cytc | 1.2128±0.0127 | | 5.6848±0.0378 | 0.9985 | 4.71±0.01 |
|  | ZnS/MR-1(sterile)/cytc | 1.3962±0.0153 | | 5.8443±0.0447 | 0.9985 | 4.76±0.04 |
|  | cytc | 1.3291±0.0135 | | 6.6114±0.1216 | 0.9955 | 4.33±0.09 |

**References**

1. Bardeen, J.; Shockley, W., Deformation Potentials and Mobilities in Non-Polar Crystals. *Physical Review* **1950,** *80*, (1), 72-80.

2. Hohenberg, P.; Kohn, W., Inhomogeneous Electron Gas. *Physical Review* **1964,** *136*, (3B), B864-B871.

3. Kohn, W.; Sham, L. J., Self-Consistent Equations Including Exchange and Correlation Effects. *Physical Review* **1965,** *140*, (4A), A1133-A1138.

4. Kresse, G.; Furthmüller, J., Efficiency of ab-initio total energy calculations for metals and semiconductors using a plane-wave basis set. *Computational Materials Science* **1996,** *6*, (1), 15-50.

5. Perdew, J. P.; Burke, K.; Ernzerhof, M., Generalized Gradient Approximation Made Simple. *Physical Review Letters* **1996,** *77*, (18), 3865-3868.

6. Monkhorst, H. J.; Pack, J. D., Special points for Brillouin-zone integrations. *Physical Review B* **1976,** *13*, (12), 5188-5192.

7. Wang, V.; Xu, N.; Liu, J.-C.; Tang, G.; Geng, W.-T., VASPKIT: A user-friendly interface facilitating high-throughput computing and analysis using VASP code. *Computer Physics Communications* **2021,** *267*, 108033.
